# Corrigendum: Dissociable Neural Information Dynamics of Perceptual Integration and Differentiation during Bistable Perception

**DOI:** 10.1093/cercor/bhaa113

**Published:** 2020-04-27

**Authors:** Andrés Canales-Johnson, Alexander J Billig, Francisco Olivares, Andrés Gonzalez, María del Carmen Garcia, Walter Silva, Esteban Vaucheret, Carlos Ciraolo, Ezequiel Mikulan, Agustín Ibanez, David Huepe, Valdas Noreika, Srivas Chennu, Tristan A Bekinschtein

**Affiliations:** 1 Department of Psychology, University of Cambridge, CB2 3EB Cambridge, UK; 2 Vicerectoria de Investigacion y Posgrado, Universidad Catolica del Maule, Talca 3480112, Chile; 3 Brain and Mind Institute, University of Western Ontario, London, N6A 3K7, Canada; 4 UCL Ear Institute, University College London, London, UK; 5 Facultad de Psicologia, Universidad Diego Portales, Santiago 8370076, Chile; 6 Programa de Cirugía de Epilepsia, Hospital Italiano de Buenos Aires, Buenos Aires C1199ABB, Argentina; 7 Laboratory of Experimental Psychology and Neuroscience (LPEN), Institute of Cognitive and Translational Neuroscience (INCyT), INECO Foundation, Favaloro University, Buenos Aires 1126, Argentina; 8 National Scientific and Technical Research Council (CONICET), Buenos Aires, Argentina; 9 School of Psychology, Center for Social and Cognitive Neuroscience (CSCN), Universidad Adolfo Ibáñez, Santiago 2485, Chile; 10 School of Computing, University of Kent, ME4 4AG Chatham, UK; 11 Department of Clinical Neurosciences, University of Cambridge, CB2 3EB Cambridge, UK

During the editing process, the name of one of the authors was mistakenly omitted.

Valdas Noreika of the Department of Psychology, University of Cambridge is the twelfth author of this article, as shown above. The authors apologize for this error.

